# Accurate Smartphone Indoor Visual Positioning Based on a High-Precision 3D Photorealistic Map

**DOI:** 10.3390/s18061974

**Published:** 2018-06-20

**Authors:** Teng Wu, Jingbin Liu, Zheng Li, Keke Liu, Beini Xu

**Affiliations:** 1 State Key Laboratory of Information Engineering in Surveying, Mapping and Remote Sensing, Wuhan University, Wuhan 430079, China; whurswuteng@whu.edu.cn (T.W.); 2014301610276@whu.edu.cn (K.L.); xubeini@whu.edu.cn (B.X.); 2 Collaborative Innovation Center of Geospatial Technology, Wuhan University, Wuhan 430079, China; 3 Chinese Antarctic Center of Surveying and Mapping, Wuhan University, Wuhan 430079, China; 2016206440005@whu.edu.cn; 4 Department of Remote Sensing and Photogrammetry and the Center of Excellence in Laser Scanning Research, Finnish Geospatial Research Institute, 02430 Masala, Finland

**Keywords:** indoor visual positioning, photogrammetric vision, place recognition, image feature matching, smartphone positioning

## Abstract

Indoor positioning is in high demand in a variety of applications, and indoor environment is a challenging scene for visual positioning. This paper proposes an accurate visual positioning method for smartphones. The proposed method includes three procedures. First, an indoor high-precision 3D photorealistic map is produced using a mobile mapping system, and the intrinsic and extrinsic parameters of the images are obtained from the mapping result. A point cloud is calculated using feature matching and multi-view forward intersection. Second, top-K similar images are queried using hamming embedding with SIFT feature description. Feature matching and pose voting are used to select correctly matched image, and the relationship between image points and 3D points is obtained. Finally, outlier points are removed using P3P with the coarse focal length. Perspective-four-point with unknown focal length and random sample consensus are used to calculate the intrinsic and extrinsic parameters of the query image and then to obtain the positioning of the smartphone. Compared with established baseline methods, the proposed method is more accurate and reliable. The experiment results show that 70 percent of the images achieve location error smaller than 0.9 m in a 10 m × 15.8 m room, and the prospect of improvement is discussed.

## 1. Introduction

With the development of smartphones and web Geographic Information System (GIS), location-based services (LBS) are changing people’s daily lives. For people in an indoor environment, indoor positioning is required. There are many technical approaches of smartphone indoor positioning [1], including inertial navigation system (INS)-based [2], WiFi-based [3], and Bluetooth-based [4] methods, but these methods have low accuracy [1], for example INS drifts along the time. To obtain high accuracy, such as less than 1 m, is expensive respect to the image based localization algorithms [5].

Mobile mapping technology has resulted in different indoor mobile mapping systems, which can produce effectively indoor high-precision three dimensional (3D) photorealistic maps, consisting of a 3D point cloud and images with known pose parameters. A 3D photorealistic map is one of the indoor spatial data infrastructures for indoor space digitalization and provides various LBSs. Cameras are also widely available in smartphones, mobile robots or wearable devices. Using an existing indoor 3D photorealistic map and camera sensor, a visual indoor positioning solution is zero-cost, and of comparatively high precision. 

In photogrammetric vision, the most popular positioning methods, i.e., structure from motion (SfM) [6,7] and simultaneous location and mapping (SLAM) [8,9] methods, are relative localization methods, and provide a high accuracy image location. This study proposes an indoor visual positioning solution which matches smartphone camera image with high-precision 3D photorealistic map. The proposed visual positioning solution is an absolute localization on a global scale, given the used photorealistic map is aligned with a global coordinate reference. The advantages of proposed absolute localization include: (1) sharing the infrastructure of an indoor map between different positioning systems; (2) absolute positioning on a global scale is required by many LBSs [10], such as iParking [11]. Indoor/outdoor seamless positioning is achieved by integrating multiple positioning techniques, for example, global navigation satellite system positioning and SLAM [12]. High accuracy is needed for some applications, such as indoor automatic moving robots. Visual-based methods are an effective method with prior GIS information. Matching light detection and ranging (LiDAR) point clouds with 2D digital line graphs are used to obtain the indoor positioning of mobile unmanned ground vehicles [13]. 

Image-based positioning has been the focus of research in the outdoors. These methods usually contain two steps, namely, place recognition [14] and perspective-n-point(PnP) [15], which calculates the extrinsic parameters. Place recognition is an important procedure. Many studies have focused on place recognition, and some benchmark datasets exist, for example, the INRIA Holidays dataset [16], Google Street View [17], and the San Francisco Landmark Dataset [18]. These methods can be classified into two categories, i.e., machine learning based and visual words based methods. Machine learning is widely used in place recognition, such as support vector machine and deep learning [19,20]. With the development of convolutional neural networks, end to end methods have been used to obtain the positioning of images, for example, PoseNet [21] and PlaNet [22]. However the accuracy of end to end based methods are lower than that of geometry based methods [23]. Supervised based methods also require many training datasets to train the parameters. 

Visual words-based methods including bag of visual words (BOW) [24], vector of locally aggregated descriptors (VALD) [25,26], and Fisher vector (FV) [27] are widely used in place recognition, especially BOW [28]. To improve the recognition efficiency, binary VALD is used for place recognition [26]. Hamming embedding is used to increase the recognition accuracy, and the angle and scale of feature points are also used to assess weak geometric consistency [16]. The automatic adjustment of parameters in the hamming embedding can improve its accuracy [29]. To handle the repetition structure of a building, geometry voting based is used to decrease the geometric burstiness [30]. Matching images with the feature point cloud instead of database images improves the efficiency of the localization procedure [31].

Most of these methods are used in outdoor environments. Due to the lack of texture, indoor scenes are more difficult than outdoor environments. To obtain global positioning, a reference database is needed [32]. Coded reference labels on walls are used to locate indoor images. These methods obtain a high accuracy from decimeters to meters [33]. The coded labels are needed to be measured by total station, and not convenient to be used. SFM method sometime fails when there is not enough feature [23], so SLAM with geometry information is used to collect large scale indoor dataset [34]. With the development of LiDAR and camera sensors, obtaining a high-precision 3D photorealistic map has become easier [34]. Backpacks with LiDAR and cameras are used to obtain large scale reference images using LiDAR based SLAM, only 55 percent of the images have a location error less than 1 m [35]. Kinect is also used to obtain the indoor visual database of the image positioning using SLAM with depth information. Then use the same data with the database, i.e., Kinect data, as query images, obtain an average location error in 0.9 m [36]. RGB and distance (RGBD) images are generated from LiDAR and image data, and these RGBD images are used as a database, then a 3D model based place recognition algorithm [37] is used to recognize the best image, and extrinsic parameters are calculated using direct linear transformation with the calibrated intrinsic parameters. The result shows a large root mean square error(RMSE) in dataset 3 in Y direction from 10 query images, i.e., 2.13 m, due to lack of features [38].

In this paper, using large scale indoor images with known pose parameters as a database, an automatic and robust visual positioning method is proposed. Place recognition and perspective-n-point (PnP) are used to calculate the pose parameters of images from smartphones. The method uses the Navvis M3 trolley [39] data as a geographic reference. The images are used as a database. Due to the lack of texture in indoor environments, appropriate image selecting in place recognition is difficult, and a posture voting to select images method is proposed. Because the focal length of the camera in a smartphone may change when capturing images [40], perspective-four-point with unknown focal length (P4Pf) [41] and random sample consensus (RANSAC) [42] are used to calculate the intrinsic and extrinsic parameters of the query image. Due to the coarse focal length can be obtained from the exchangeable image file format (EXIF) information, perspective-three-point (P3P) [43] is used to remove the outlier points. A large number of images are used to prove the effectiveness of the proposed method. The remainder of the paper is structured as follows: Section 2 formulates the proposed visual positioning method. Section 3 describes the experimental analysis. Section 4 is the discussion. Section 5 is the conclusion. 

## 2. Methodology

The proposed method uses the images from the vehicle survey data captured by a Navvis M3 trolley as the database images. The query images are from smartphones. By registering with the database images, the position of the current image from the smartphone is obtained.

### 2.1. High-Precision 3D Photorealistic Map

Using SfM to calculate the intrinsic and extrinsic parameters of images is commonly used in richly textured environments [44]. In an indoor environment, failure occurs due to the lack of texture and repetition structures [23]. LiDAR is a new sensor to range objects in a real-world scale, and 2D LiDAR SLAM is widely used in indoor environments [45]. Navvis M3 trolley [39] is a map producer based on the TUMindoor [34]. There are three Hokuyo laser range finders and six Panasonic DMC-GX7 cameras with 16-Megapixels. Through 2D LiDAR SLAM, the platform calculates the pose parameters of the full trajectory. The relative parameters of cameras are calibrated, through similarity transformation, and the extrinsic parameters of the image are obtained. The intrinsic parameters of the cameras are also calibrated. The dataset constituted by the images with known intrinsic and extrinsic parameters is called a high-precision 3D photorealistic map. After collecting the high-precision 3D photorealistic map, images are used as database images for smartphone visual positioning. After the high-precision 3D photorealistic map is produced by the trolley, there are two parts in the method, i.e., database image processing and smartphone image positioning.

### 2.2. Database Image Processing

**Figure 1:**
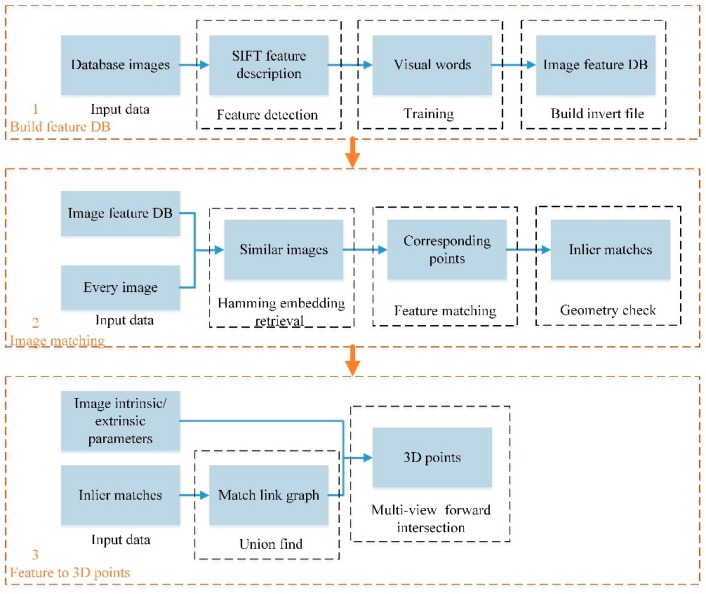
Workflow of database Image processing.

The goal of database image processing is to build the feature database (DB) for place recognition and calculate the 3D points for the PnP method. The workflow is shown in the Figure 1. 

#### 2.2.1. Build Feature Database

Due to the lack of texture, the indoor environment is not well suited for image matching, and selecting reliable method is important. In feature matching, instead of matching all the images in the database, image retrieval can improve the computer efficiency and only match the similar images. A feature database is needed in the image retrieval. Feature point detector and SIFT description are used for feature extraction. RootSIFT which is easy to implement, is better than SIFT in image retrieval [46], so the SIFT description is converted into RootSIFT. In image retrieval, hamming embedding outperforms BOW [29], so hamming embedding is used in the procedure.

Before building an inverted file, a visual word vocabulary is needed [16]. In order to improve the computer efficiency of clustering, hierarchical k-means is used to train the visual words [47]. After training, the hamming embedding method is used to build the inverted file [29].

#### 2.2.2. Image Feature Matching

Image feature matching is a time-cost procedure when the number of image is large. A coarse to fine matching method is utilized to improve the computing efficiency [47], instead of matching all the images in the database, only top similar images are matched, i.e., top 100. First, similar images is identified using the hamming embedding method [29]. 

**Figure 2:**
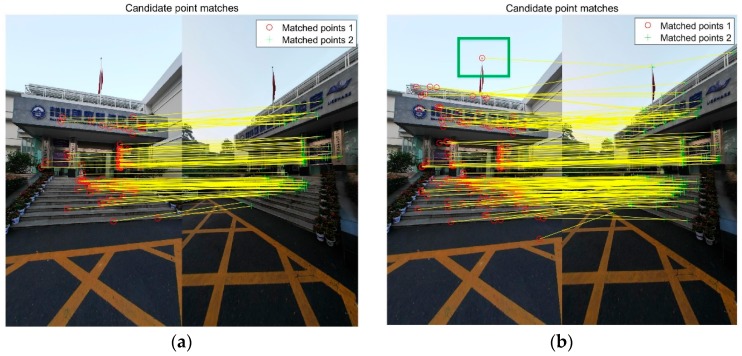
Comparison between the F matrix and E matrix in geometry check. (**a**) RANSAC and fundamental matrix as geometry check, 123 points; (**b**) Calculate essential matrix from extrinsic parameters as geometry check, 219 points.

For every image, after the high similarity images are queried, the line of sight of the images are used to remove useless matches. Second, feature description matching is used to match image pairs. There is noise in the feature match pair. Thus, RANSAC and fundamental matrix geometry check were used to remove the outliers. In the experiment, the poses of the database images can be calculated, the absolute pose is used to get the essential matrix, and a geometry check removes the outliers. Essential matrix (EM) based is faster and can obtain more correct points than RANSAC and fundamental matrix (FM) based. In the experiment, the CPU is an Intel Corei7-6700HQ with 16 GB memory. The computational time of FM method is 0.031 s with 1000 times in RANSAC, and the computational time of EM method is less than 1 ms. The result is shown in Figure 2, the method calculates the essential matrix from extrinsic parameters, as the geometry check obtains more points, the point on the flagpole is matched, the green rectangle.

#### 2.2.3. Feature Points to 3D Point Cloud

**Figure 3:**
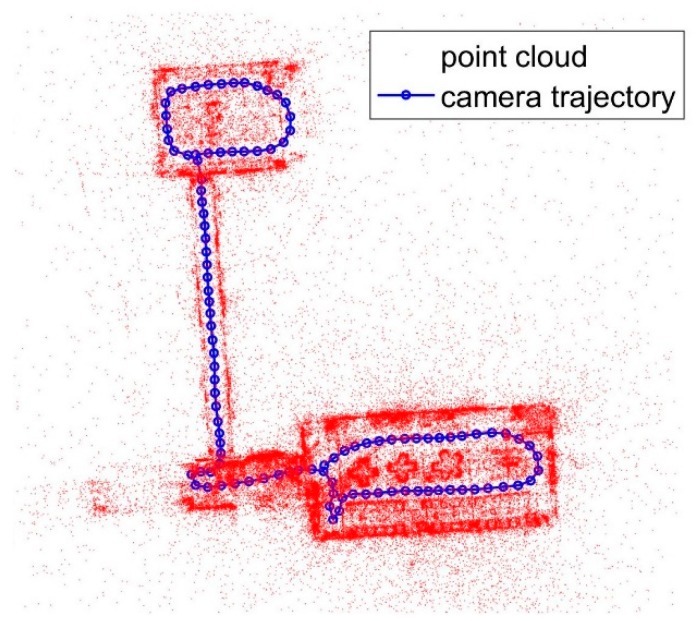
Point cloud from multi-view forward intersection.

The poses of the images from the database can be calculated using 2D LiDAR SLAM results and the calibration parameters. These images are used as database images. Then, the images are used to obtain the point cloud. Using feature matching and forward intersection is a common way to obtain 3D points. After feature matching, the union-find approach is used to link the feature points from image to image [48]. Some points may have more than two observations from the images. Multi-view forward intersection is adopted, because some observations are outliers, so RANSAC and forward intersection are used. As shown in Figure 3, the red points are the feature point cloud, the blue points are the camera exposure location and the line is the trolley trajectory. After the geometry check, there are many outliers in the point cloud.

### 2.3. Smartphone Visual Positioning

**Figure 4:**
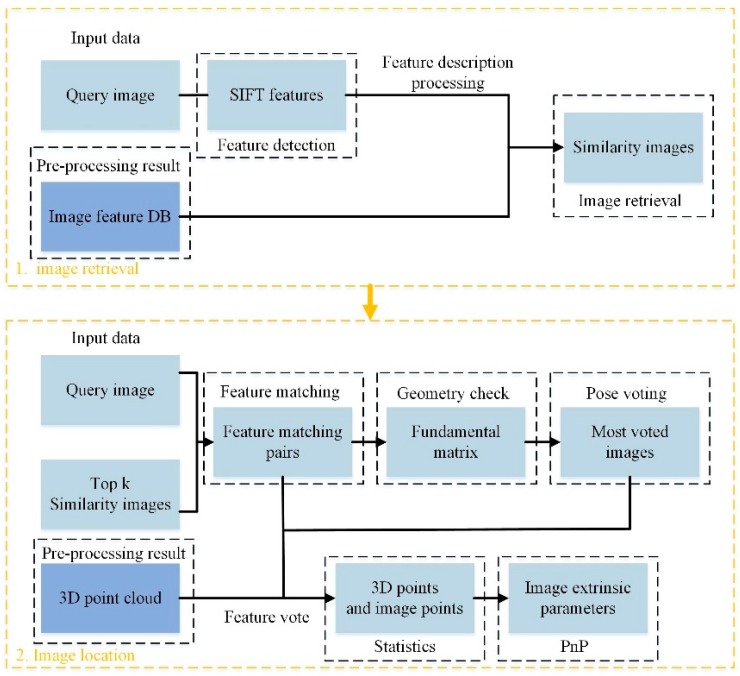
Workflow of smartphone image positioning.

The focal length of the smartphone cameras may change during autofocus when capturing images [40]. Because camera calibration is not familiar and difficult to unprofessional users, the camera is not calibrated before capturing images, and this can improve the usability of the proposed method. The smartphone image positioning procedure is shown in Figure 4. The data in the deep blue rectangle is from preprocessing database images.

To get the positioning of the query image, enough 3D points should be matched to calculate the extrinsic parameters. P4Pf is used in the method, and pose is estimated when more than 12 points are matched in the experiment. The database images and query images are from different sensors. The exposure environment changes, and the scale of the images varies. It is quite difficult to match the query images and the database images.

The BOW-based place recognition method was used to obtain the top similar images, which sometimes contains a similar scene, and there are few match points. The database includes some image containing the same scene, and more image are queried to obtain a stable result. A pose voting method is used to select the images.

#### 2.3.1. Place Recognition and Feature Matching

Place recognition is an important procedure in visual positioning. Hamming embedding [29] is used in the experiment. The most popular feature point detectors used in place recognition are Hessian affine and difference of Gaussians (DoG) feature detectors.

In two pair feature matching, a DoG detector was shown to be better [49]. But in indoor place recognition, few works have produced this comparison. In this paper, 930 images collected by the trolley, and 130 images captured by smartphones were used. The database images have a large overlap, so the query image often has many correct corresponding images. The mean average precision (mAP) was used to evaluate the place recognition accuracy. In computing the average precision, the positive images are the images that have at least 50 percent overlap with the query image. The negative images do not have 50 percent overlap. The number of correct images makes a difference on the stability of the results in the experiment. 

**Figure 5:**
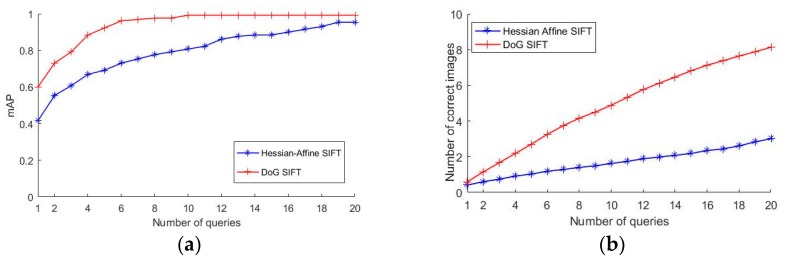
Comparison between Hessian affine and DoG detectors. (**a**) mAP (**b**) average number of correct images in the top-K queries.

As shown in Figure 5, the experiment shows that the DoG detector outperformed Hessian affine detector. The mAP of the top five query results using the DoG detector reached 0.923, while the mAP was 0.692 using the Hessian affine detector in the experiment. The DoG detector was used in the next experiment. In the place recognition procedure, the DoG features are extracted in the query image. Visual word matching and score sorting obtain the query result. 

After the query procedure, feature matching using minimum distance and geometry check using fundamental matrix and RANSAC are used to select the best match image in the top-K images. In the database images, the same place is visible in several images. 

**Figure 6:**
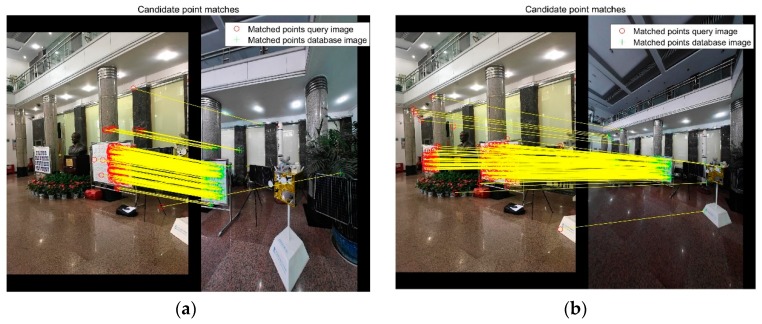
Matches after geometry check. (**a**) Most matched image with 390 matches. (**b**) A uniform distribution with 339 matches.

In the experiment, the most matched image is not always the best image to computer the pose of the query image. Figure 6 shows that, the image in the left figure has more feature matches, but the distribution is not very good. The correct feature points occur approximately in a regional area. The image in the right figure has a good distribution match result. To improve the stability of the method, a voting based method is proposed in smartphone image place recognition.

#### 2.3.2. Voting-Based Image Selection

SIFT-based feature matching using minimum distance is adopted to match the top-K images in the query result. After feature matching, RANSAC and a fundamental matrix based geometry check are applied to remove the outlier matches. The different resources and points of view of the images make the feature matching difficult, so the correct matches are only a small ratio in the queried matches. When the query image contains many non-textured areas, there are few correct match points. Selecting more than one image make the result stable. The Euler angles are decomposed from the rotation matrix of the query image, i.e., ϕ, ω and κ. In the voting result, the most supported value is correct. An angle based vote method is adopted to select the correct image. 

RANSAC and a fundamental matrix based geometry check are used to obtain the most voted fundamental matrix. The intrinsic parameters of the database images are known, and from the exchangeable image file (EXIF) from the smartphone image file, the coarse focal length is obtained, and the distortion parameters of the query image are set to zero. A coarse *K* matrix of the query image is composed, as shown in Equation (1). *F* is the fundamental matrix, and *E* is the essential matrix:

(1) $$E={K^{\prime }}_{1}\cdot F\cdot K_{0}$$

After calculating the essential matrix, the decomposed matrix obtains the relative rotation and translation as shown in Equation (2), *R_LR_* is the relative rotation matrix, and [*t*]_x_ is the symmetric matrix of the translation vector:

(2) $$E={\left[t\right]}_{x}\cdot R_{LR}$$

The database images had extrinsic parameters, so the absolute rotation matrix was calculated. Through the relative rotation, the coarse rotation matrix is obtained, as shown in Equation (3), *R_L_* is the rotation matrix of the image from database, and *R_R_* is the rotation matrix of the query image:

(3) $$R_{R}=R_{L}\cdot R_{LR}$$

After obtaining the rotation matrix, the Euler angles can be calculated, as shown in Equation (4), *R_ij_* is the element of *i* row and *j* column:

(4) $$\begin{array}{l} \phi =\mathrm{atan}2\left(-R_{13},R_{33}\right)\\ \omega =\mathrm{asin}\left(-R_{13}\right)\\ \kappa =\mathrm{atan}2\left(-R_{21},R_{22}\right) \end{array}$$

The angles are in a 3D parameter space, and the space is partitioned into 3D cubes, for example the size of a cube is 15 × 15 × 15 degrees, so that 24 × 24 × 24 cubes are obtained. After all the images are calculated, the correct images will aggregate in a cube, the most supported Euler angles are obtained, also the corresponding matched images are the selected images.

**Figure 7:**
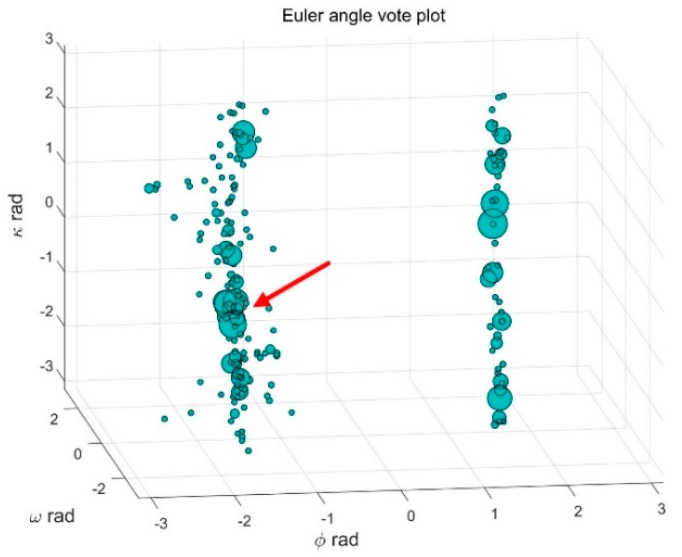
Euler angle vote plot.

**Figure 8:**
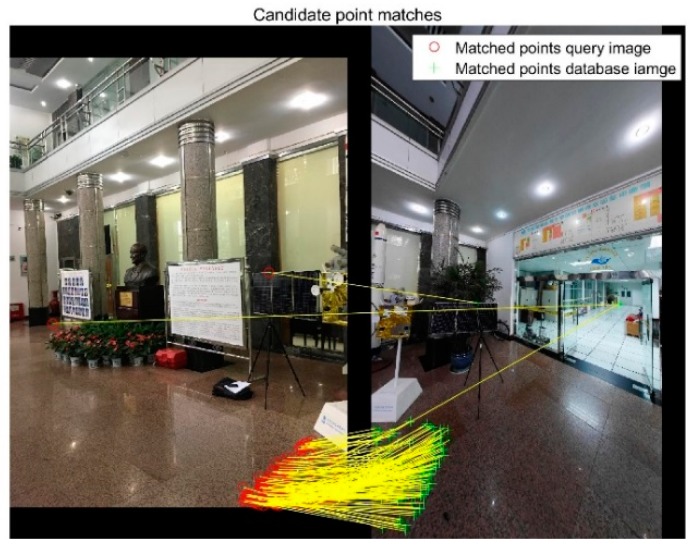
Bad match image with 182 matches after geometry check.

A vote result of an image is shown in Figure 7. The size of the point represents the number of matches after the geometry check, the points aggregate at the correct pose, as the red arrow in Figure 7. After voting, several images are selected, and bad matches are removed. Even though the number is large, as shown in Figure 8, the image is removed after voting.

#### 2.3.3. Image Positioning

Usually, several images are selected in the voting procedure. The features of the query image usually correspond to several images. Using the relationship between the feature points and 3D points, the 3D points of the feature points on the query image are obtained. After multi-view forward intersection, the relation between the 3D points and the feature points of the database images is built. Through the matching feature points, the corresponding 3D points of the feature points from the query image are obtained. Then, perspective-n-point (PnP) based method is used to calculate the extrinsic parameters of the query image. 

To remove some outliers, RANSAC [42], and P3P [43] with the coarse focal length from the EXIF file in smartphone images are used to remove the outliers in the 2D–3D points. The cameras autofocus during exposure. The intrinsic parameters also change after calibration. The smartphone camera is not calibrated before capturing the images, and the focal length is calculated in the PnP procedure. Perspective-four-point with unknown focal length (P4Pf) [41] is adopted. Because there exist error matches, RANSAC is combined with the estimator. Perspective-five-point calculates the radial distortion parameters (P5Pfr) [50] is studied in the experiment and ensure the stability of the P5Pfr result, only the first distortion parameter (k1) is calculated. 

In Table 1, the f is the focal length, k1 is the distortion parameter, and Δd is the total error from the ground-truth point. From the EXIF information, the coarse focal length is 3278 in pixel. Table 1 shows that using P5Pfr the result is different from this value and differs greatly from image to image. The correct radial distortion parameter is small. In image 3, there are many outliers in the 2D-3D points, so the result of P5Pfr is worse, and P4Pf obtains a stable result. Therefore, in the experiment, P4Pf is used to calculate the camera pose.

## 3. Experimental Analysis

### 3.1. Test Data

In the experiment, the database images are from the Navvis M3 trolley [39]. There are three laser range finders, and six cameras mounted on the trolley. The database images are obtained from Panasonic cameras with fisheye lenses. The positioning information is obtained using 2D SLAM based on the horizontal LiDAR sensor. The intrinsic and relative extrinsic parameters of the cameras are obtained from calibration. The pose parameters of the database images are calculated from spatial transformation between the camera coordinated system and the LiDAR coordinated system.

**Figure 9:**
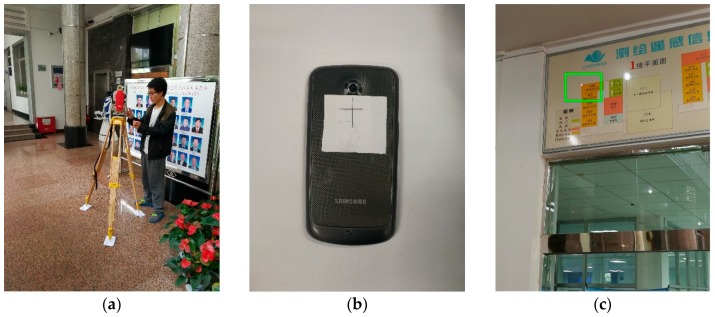
(**a**) The total station; (**b**) The mark on the smartphone; (**c**) The control point measured using the total station.

To evaluate the precision of the positioning, the non-prism total station is used to measure the camera positioning on the smartphone in the experiment. In the experiment, it is difficult to measure the camera on the smartphone because the surface of the camera is glass material. A particular mark is made on the smartphone, for example, a crosshair, as shown in Figure 9b. The person walks around in the room, and then the person hold the smartphone still to capture an image, and use the total station to measure the crosshair on the phone. After measuring the offset, then the camera position is acquired. Some common points on the wall are measured to calculate the spatial transformation parameters between the database images coordinated system and the smartphone images coordinated system, as shown in Figure 9c. The ground truth coordinates measured by the total station are converted to the database image coordinate system using spatial transformation.

In the experiment, two smart phones are used to capture the query images, a Samsung Galaxy Nexus and an Apple iPhone 6s. The pixel size of the Samsung Galaxy Nexus is 1.4 μm, and the image size is 1944 × 2592. The pixel size of the Apple iPhone 6s is 1.22 μm and the image size is 3024 × 4032. According to the pixel size information and the EXIF information in the JPG file, the coarse focal length is calculated. 

### 3.2. Accuracy Evaluation

To prove the effectiveness of the proposed method, the baseline is the original method using the image with the most matches to calculate the extrinsic parameters of the smartphone camera as in [35]. To evaluate the proposed method, two different places are used in the experiment. In Table 2 and Table 3, RMSE is the root mean square error, and LE90 is the linear error with 90% confidence. Δ*X*, Δ*Y*, Δ*Z* are the three error components, and Δ*d* is the total error from the ground-truth points.

**Figure 10:**
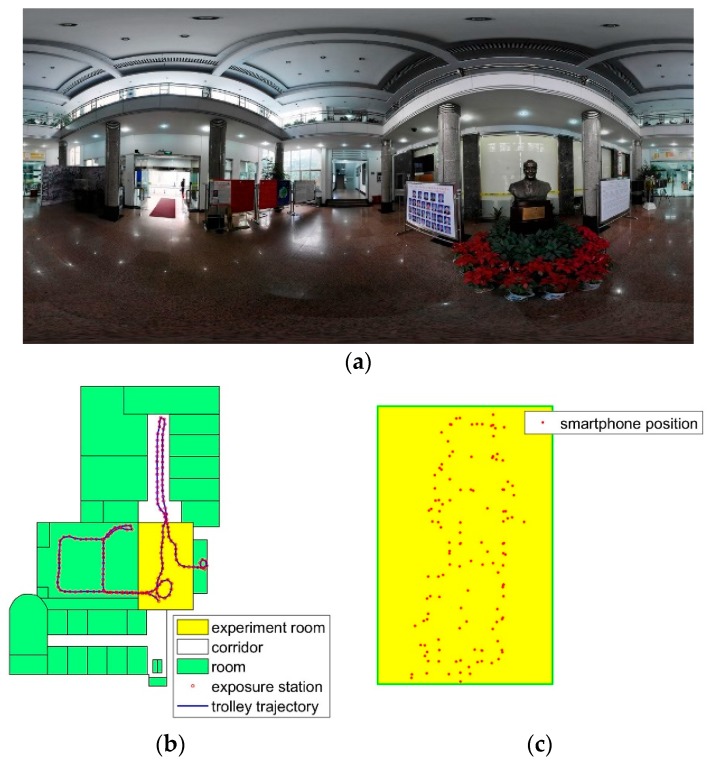
Data description in experiment one. (**a**) Panorama image of the room. (**b**) Database image data description. (**c**) The smartphone positions in the experiment room.

One dataset is collected from a hall in the laboratory. The panorama image is shown in Figure 10a. The database images are shown as red rings in Figure 10b. The trolley trajectory is the M3 scanning path, and the red rings are the exposure positions. In Figure 10c, the red dots are the smartphone positions where the person stands still to capture an image. In the experiment, the query images are from four stations. The database contains 930 images, and the whole scanned space is about 408 m^2^ in ground surface. The experiment room size is 10 m × 15.8 m in ground surface. The room contains little furniture, and the images are captured from different viewpoints and positions. In the experiment, many query images are similar, as shown in Figure 10c, the smartphone points have a high density in the room. 

In the experiment, if the number of feature points is less than six, the query image is invalid. The total number of images from the Samsung is 78. Referring to Table 2, there are 72 images in the proposed method, and 71 in the baseline method. There are 43 valid images in total 51 images in the iPhone dataset. When selecting several images to obtain the 2D–3D points, more points are obtained. On the RMSE metric, the proposed method result is more accurate. When calculating the extrinsic parameters, the outlier points have a great difference on the result. Selecting more images can improve the inlier ratio in the experiment. More stable positioning result is reached, as shown in Table 2, the positioning error of 90 percent of the images is less than that of the baseline method.

**Figure 11:**
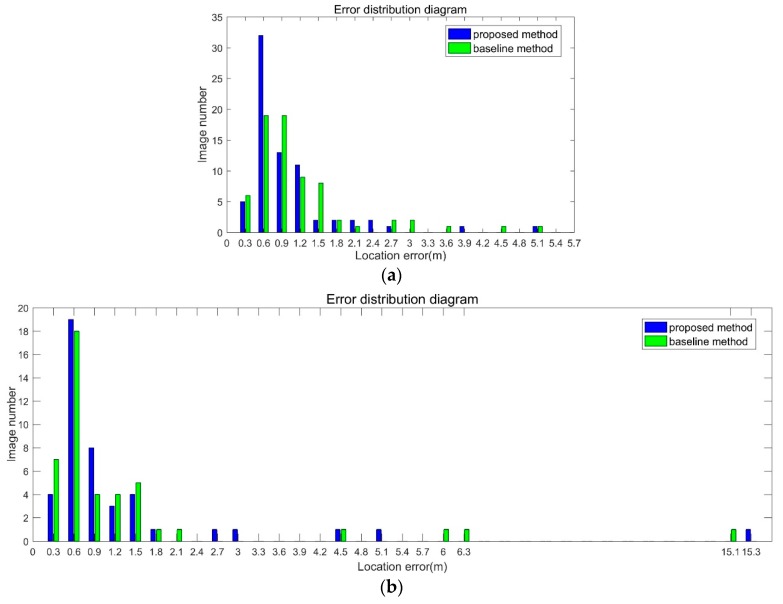
The location error distribution diagram of dataset 1. (**a**) Samsung dataset result. (**b**) iPhone dataset result.

Indoor environment is very challenging for visual matching. The position error in not uniform distributed, as shown in Figure 11. The *X* axis in the figure is the location error in the range, for example, 0–0.3, 0.3–0.6, 0.6–0.9 and so on. The *Y* axis is the image number.

As shown in Figure 11a, location error of most images is small. The location error of 70 percent of the images in the proposed method is smaller than 0.9 m, and the baseline is 62 percent. The max error is 4.9 m. In Figure 11b, the error distribution is divergent. There exist gross errors, for example 6.3 m and 15.1 m. The location error of 70 percent of the images in the proposed method is smaller than 0.9 m, and the baseline is 66 percent. The divergent errors bring large value in RMSE.

**Figure 12:**
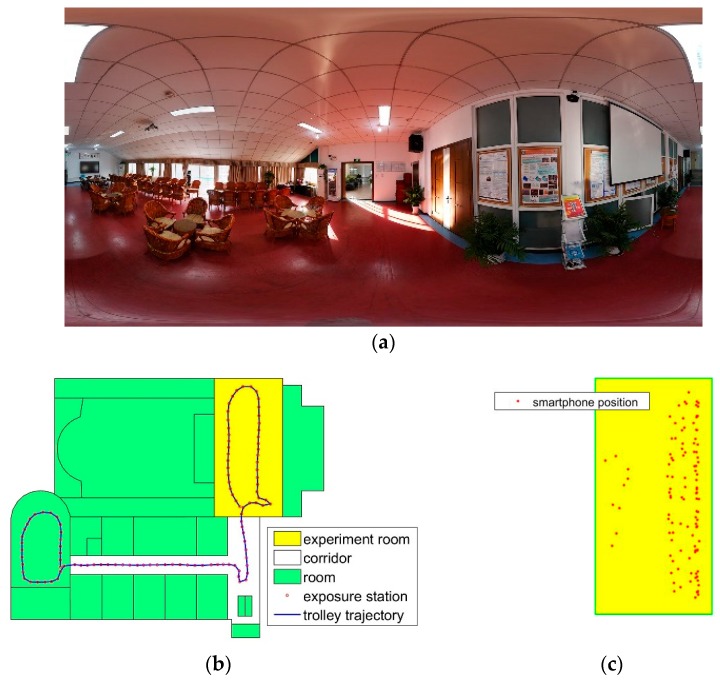
Data description in a challenging scene. (**a**) Panorama image of the room. (**b**) Database image data description. (**c**) The smartphone positions in the experiment room.

Another dataset is collected in a multipurpose room. The query images are from three stations, the panorama image is shown in Figure 12a, and there are many repetition structures in the room. The feature points are fewer than dataset 1 and non-uniform distributed. The database images are shown in Figure 12b. In Figure 12c, the red dots are the smartphone positions where the person stands still to capture an image. There are 642 images in the database, and the whole scanned space is about 223 m^2^ in ground surface. The experiment room size is 7.8 m × 15.8 m in ground surface, and 55 images are taken in the challenge environment for each smartphone. Because the wall and floor have no texture, only one wall with posters contains some feature points, and the images are all captured from the wall. 

Reference to Table 3, there are 41 valid of total 55 images in the Samsung dataset. In iPhone dataset, there are 78 images in the proposed method, and 47 in the baseline method. This scene is a more challenging environment. The position error is more divergent. On the RMSE metric, the proposed method result is more accurate. In the experiment, the feature points gather on the posters and the match points are unevenly distributed. Selecting more images can improve the position stability, as shown in Table 3, the error of L90 is less than baseline method. In the experiment, the resolution changes between the database images, and the iPhone images have a high resolution, and the image scale is more close to that of the database images. There are few feature points in the scene, and there are more feature points detected in the iPhone image, so the iPhone images obtain better accuracy. 

**Figure 13:**
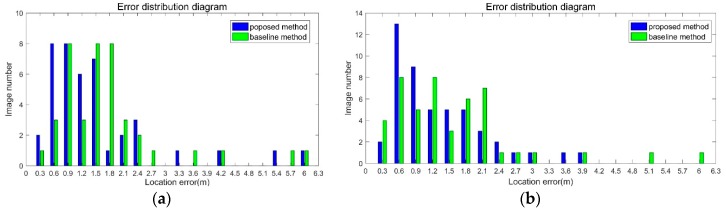
The location error distribution diagram of dataset 2. (**a**) Samsung dataset result. (**b**) iPhone dataset result.

As shown in Figure 13a, the location error of 43 percent of the images in the proposed method is smaller than 0.9m, and the baseline is 29 percent. The max error is 5.6 m. In Figure 13b, the location error of 50 percent of the images in the proposed method is smaller than 0.9m, and the baseline is 36 percent. In this scene, the distribution of feature points is non-uniform. This bring large error in PnP estimator. The divergent errors bring large value in RMSE.

## 4. Discussion

Indoor environments are challenging for visual positioning because there are weak texture regions or textureless regions. In the weak texture scenes, there is no result, for example 15 in 159 images could not be matched in Table 2.

In the method that relies on the 3D photorealistic map, the accuracy of the image database has a great influence on the positioning result on the smartphones. In the experiment, the 2D pose results are from 2D LiDAR SLAM. The pose parameters of the image are calculated using the SLAM result and calibration information. The error is accumulated with the trolley moving. The accumulation error is large in the scanning trajectory, resulting in the inconsistency of the extrinsic parameters along different positions because of error propagation. In the multiview intersection, the intersection error of the control points on the image is big. The error has a great difference on the forward intersection result. In the experiment, the 3D coordinates in image database space of the control points on the wall, as shown in Figure 9c, are using the forward intersection. The reprojection error is big when the exposure station is far away from each other. The min error is 1.0 pixel, and the max error is 43.3 pixel, as shown in Table 4. The exposure locations of cameras are discontinuous, for example, 1.5 m one time. In the experiment, the distance is 0.8 m in average. The first row is the exposure station index, the second row is the reprojection error in pixel, and the third row is the multi-view forward intersection result in meter. The number headings 14-0 and 14-1 are the two images at the same station. The standard deviation of the errors is 15.96. If the exposure station is adjacent, the error is coincident, for example from 13 to 17, reprojection errors are all small, on the other hand, form 61 to 68, reprojection errors are all large. It is a long way for the trolley to move from station 17 to station 61, and the error accumulation is big, which brings error to the pose parameters of database images. As shown in Table 4, the fourth row is the reprojection errors if separating the measurements into two parts, i.e., from 13 to 17 are group 1 and from 61 to 68 are group 2. The last row is the forward intersection result of each group. The difference of the results is 0.14 m.

After obtaining the coordinates of the control points in image database space, the spatial transformation can be calculated. The optimization in the spatial transformation adjustment does not converge [51], because the error is gross. As shown in Table 5, the average error is about 0.037 m, and the max error is 0.071 m.

**Figure 14:**
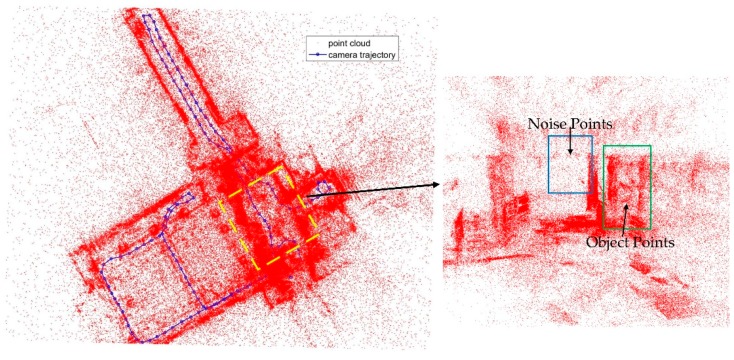
Noise points in the feature 3D points.

In the feature matching, because there are repetition features, and the number of overlap images is small, mostly 2 or 3 in the experiment, so there are many noise points in the feature point cloud. As shown in Figure 14, there are some wrong points in the point cloud, mainly the low density points in the blue rectangle. This will bring errors to positioning result [52].

In the PnP pose estimator, only focal length is calculated. The principal point and distortion parameters are not solved in P4Pf. An experiment with fixed focal length is adopted to analyze the error. Before capturing the images, the camera is calibrated.

As shown in Table 6, in general, calibration of camera can improve the accuracy. The position error of 8 images is smaller than 0.6 m after calibration. The PnP methods are both sensitive to noise, for example image 3, 8, 9. On the other hand, the distribution of feature points has effect on the result.

After calibration, the focal length is obtained, i.e., f_x_ is 3365.81 and f_y_ is 3379.74 in pixel. As listed in Table 6, the gross errors occur when the focal length is far away from the calibration result, for example image 2, 4 and 9. The difference is larger than 800. This is a useful threshold in RANSAC procedure. Using the constraint that the difference between the coarse length in EXIF information and the focal length of P4Pf is larger than a threshold, i.e., 800, in the experiment can improve the position accuracy. 

Even though camera calibration improves the location accuracy, this brings a great problem to unprofessional users. Camera calibration is not easy for unprofessional users. In the experiment, after exiting from the build-in photo APP, the APP will be autofocus when opened in next time. The focal length will change and the calibration is needed again.

**Figure 15:**
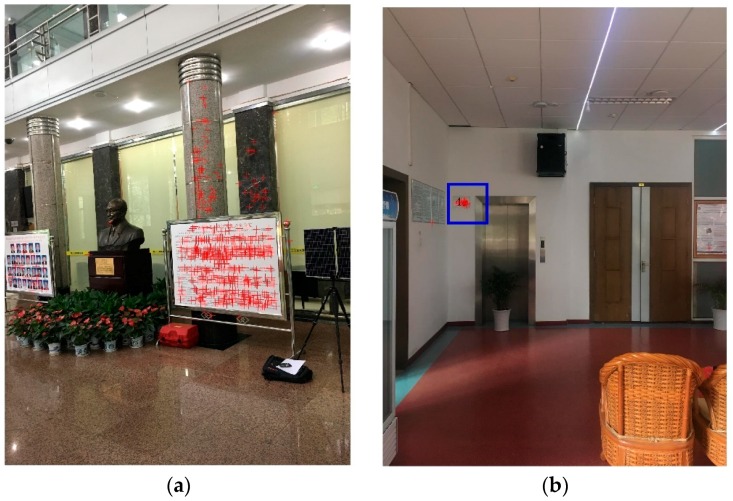
Distribution of feature points before PnP estimator. (**a**) Good distribution and enough feature points. (**b**) Bad distribution of feature points.

In the experiment, the distribution of feature points has an effect on the PnP result. Non-uniform distributed feature points give unstable pose estimates and sometime cause gross error [44]. In dataset 1, the walls contain some features, and the distribution is better than dataset 2, as shown in Figure 15. As shown in the blue rectangle in Figure 15b, the number of feature points is 23, but they gather in a small region. This brings gross error in PnP estimator.

**Figure 16:**
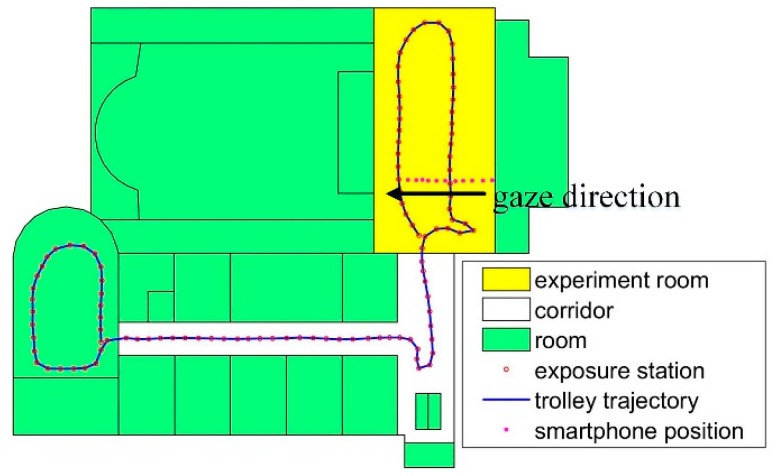
Images are collected along a line.

To analyze the effect of the distance to the objects, the images are collected in a line in the experiment, as shown in Figure 16. The distance to the wall is from 2.20 m to 7.64 m. 

There are 11 images in the test. The gaze direction is the black arrow shown in Figure 16. The result is listed in Table 7. Δ*d* is the distance error from ground-truth point. There is no relationship between the accuracy and the distance to the objects, and the feature match errors and the distribution of feature points influence the location accuracy.

**Figure 17:**
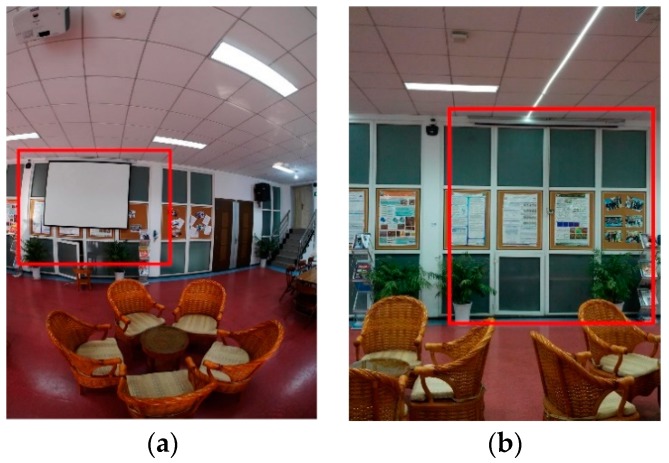
Change of the environment, in the red rectangle. (**a**) Image captured on 4 December 2016. (**b**) Image captured on 16 October 2017.

Changes in the scene also affect the method, as shown in Figure 17, and sometimes, the furniture may move frequently in the scene. Some images could not be matched because of the changes, which results in gross errors. 

The proposed method is a prototype system for smartphone indoor visual positioning. The processing of database images can be done offline, so only the computational time of processing the smartphone image is discussed following. The images are processed in the original size. The procedure is computed on the computer without parallel optimization, except the feature extraction and matching is using SiftGPU [53]. The method is implemented with Microsoft Visual C++ under the Microsoft Windows 10 operating system. A personal computer with Intel Corei7-6700HQ, 2.59 GHz CPU, NIVIDA Quadro M1000M, 4 GB GPU, 16 GB memory is used. The image size and the number of feature points make a difference on the computational time. In the experiment, five images are used and average time is listed in Table 8.

As shown in Table 8, the total time is 1–2 min, and the feature matching takes the most time. In the experiment, only top 50 similar images are matched. Using hamming embedding to find similar images also costs a lot of time. In the experiment, the number of database images is 642, and if the number is larger, more time is needed. To reduce the computational time, a coarse position can be useful when only the adjacent images are compared in query. Using downsampled images can also improve computational efficiency in engineering.

## 5. Conclusions

In this paper, an automatic and robust visual smartphone indoor positioning method is proposed, using images with known intrinsic and extrinsic parameters to locate smartphones. Using feature matching and multiview forward intersection to obtain a point cloud, and place recognition and pose voting to select similar images, the point cloud and the image points are used to calculate the intrinsic and extrinsic parameters of the query image. The experiment shows that 70 percent of the images achieve location error smaller than 0.9 m in a 10 m × 15.8 m room, and the proposed method is more stable. Even in a challenging scene, 43 percent of the Samsung images and 50 percent of the iPhone images achieve location error smaller than 0.9 m in a 7.8 m × 15.5 m room. In the paper, the location error is also analyzed.

The proposed method also has limitations. The world points are obtained from feature matching, and the relationship between the image points and the world points is obtained from feature matching. Thus, in weak texture areas, there will be no result or inaccurate positioning [54]. The distribution of feature points has a great effect on the PnP result. In future work, feature lines will be used to improve the positioning accuracy. However, the pose accuracy of the database images determines the positioning accuracy of the query images. The optimization of the pose parameters of images is also important in the positioning procedure.

## Figures and Tables

**Table 1:** Comparison between P4Pf and P5Pfr methods.

No.	Image 1			Image 2			Image 3		
Method	P4Pf	P5Pfr	Truth	P4Pf	P5Pfr	Truth	P4Pf	P5Pfr	Truth
cx (m)	−8.035	−7.961	−8.282	−6.922	−6.805	−7.173	−5.671	−6.159	−5.869
cy (m)	2.756	2.857	2.684	1.558	1.798	1.518	0.254	−2.071	0.508
cz (m)	1.609	1.610	1.672	1.604	1.641	1.672	1.561	1.405	1.666
f (pixel)	**3373.53**	**3437.09**	**----**	**3397.72**	**3563.95**	**----**	**3339.37**	**2324.98**	**----**
k1	**----**	**−0.013673**	**----**	**----**	**0.019428**	**----**	**----**	**−0.985972**	**----**
Δ*d*	**0.2649**	**0.3699**	**----**	**0.2631**	**0.4634**	**----**	**0.3387**	**2.6083**	**----**

**Table 2:** Accuracy assessment of the first dataset.

Phone (Image Number)		Samsung (78)		iPhone (51)	
Method (Valid Number)		Proposed (72)	Baseline (71)	Proposed (43)	Baseline (43)
RMSE	Δ*X* (m)	0.78	0.77	0.57	0.69
	Δ*Y* (m)	0.70	0.77	0.97	2.24
	Δ*Z* (m)	0.21	0.61	0.54	1.28
	Δ*d* (m)	**1.07**	**1.25**	**1.25**	**2.67**
LE90	Δ*X* (m)	0.85	1.03	1.02	1.21
	Δ*Y* (m)	1.02	1.02	0.87	1.17
	Δ*Z* (m)	0.40	0.40	0.40	0.41
	Δ*d* (m)	**1.61**	**1.80**	**1.40**	**1.90**

**Table 3:** Accuracy evaluation of the second dataset.

Phone (Image Number)		Samsung (55)		iPhone (55)	
Method (Valid Number)		Proposed (41)	Baseline (41)	Proposed (48)	Baseline (47)
RMSE	Δ*X* (m)	1.07	1.19	0.74	1.29
	Δ*Y* (m)	1.13	1.27	0.87	1.12
	Δ*Z* (m)	0.73	0.64	0.58	0.46
	Δ*d* (m)	**1.72**	**1.87**	**1.29**	**1.71**
LE90	Δ*X* (m)	1.44	1.50	1.41	1.74
	Δ*Y* (m)	1.63	1.78	1.48	1.61
	Δ*Z* (m)	1.08	1.01	0.75	0.87
	Δ*d* (m)	**2.13**	**2.47**	**2.07**	**2.41**

**Table 4:** Reprojection error after forward intersection with the exposure station.

Station No.	13	14-0	14-1	15	16	17	61	62	63	64	65	66	67	68
Error (pixel)	2.7	12.7	3.4	2.8	**1.0**	3.6	41.2	**43.3**	35.8	33.3	29.4	28.6	31.6	28.0
*XYZ* (m)	−12.230				1.814					3.590				
Error (pixel)	2.7	12.7	3.4	2.8	**1.0**	3.6	19.2	**2.9**	1.6	2.0	3.0	4.7	9.5	9.9
*XYZ* (m)	−12.230		1.814		3.590		−12.210			1.812			3.455	

**Table 5:** Point error in spatial transformation in bad condition.

Point No.	1	2	3	4	5	6	Average
Error (m)	0.027	0.016	0.062	**0.071**	0.030	0.017	**0.037**

**Table 6:** The influence of camera calibration.

Image NO.	Calibration (P3P + RANSAC)	Proposed (P4Pf + RANSAC)	
	Δ*d* (m)	Δ*d* (m)	Focal Length (Pixel)
1	0.51	1.07	4023.70
2	0.21	3.01	**2400.18**
3	1.72	1.07	3015.51
4	0.30	**4.78**	**2562.98**
5	0.39	1.11	4020.93
6	0.39	1.32	2831.20
7	0.67	1.24	2973.64
8	**3.23**	1.22	4224.38
9	1.47	2.09	**2308.51**
10	0.37	0.93	4094.44
11	0.23	0.65	3890.77
12	0.68	1.23	2591.89
13	**0.21**	1.41	3679.71
14	0.61	**0.62**	3211.18

**Table 7:** Analysis of images along a line.

Distance to Wall (m)	Measure			Ground-Truth			Δ*d*
	*X*	*Y*	*Z*	*X*	*Y*	*Z*	
2.20	13.090	−19.493	1.810	13.164	−19.181	1.618	0.374
2.74	13.297	−20.033	1.409	13.185	−19.721	1.620	0.393
3.14	13.098	−19.380	1.699	13.237	−20.113	1.613	0.751
3.51	13.107	−20.226	1.745	13.208	−20.494	1.612	0.316
4.05	13.091	−20.471	1.600	13.237	−21.033	1.613	0.580
4.61	13.221	−20.644	1.642	13.273	−21.597	1.602	0.955
5.27	13.579	−22.447	1.608	13.299	−22.263	1.630	0.336
5.76	13.307	−21.308	1.377	13.358	−22.756	1.632	1.471
6.35	13.413	−21.990	1.172	13.404	−23.342	1.631	1.429
7.05	13.457	−24.244	1.758	13.453	−24.045	1.637	**0.233**
7.64	12.888	−23.074	2.280	13.517	−24.638	1.704	**1.782**

**Table 8:** Computational time of each procedure.

Procedure	Samsung Image	iPhone Image
Feature extraction	<1 ms	<1 ms
Similar image query	29.539 s	44.305 s
Feature matching	**39.487** s	**65.525** s
Pose calculation	0.710 s	0.940 s
Total	69.736 s	110.770 s
